# Molecular identification and phylogenetic profiling of African swine fever virus in Indonesia during 2021–2025 based on the *B646L* (p72) gene

**DOI:** 10.14202/vetworld.2026.1437-1446

**Published:** 2026-04-12

**Authors:** Muharam Saepulloh, Ni Luh Putu Indi Dharmayanti, Aswin Rafif Khairullah, Harimurti Nuradji, Wiwiek Tyasningsih, Atik Ratnawati, Mustofa Helmi Effendi, Indrawati Sendow, Budiastuti Budiastuti, Susanti Susanti, Dian Ayu Permatasari, Saifur Rehman

**Affiliations:** 1 Research Center for Veterinary Science, National Research and Innovation Agency (BRIN), Jl. Raya Bogor Km. 46 Cibinong, Bogor 16911, West Java, Indonesia; 2 Department of Veterinary Microbiology, Faculty of Veterinary Medicine, Universitas Airlangga, Jl. Dr. Ir. H. Soekarno, Kampus C Mulyorejo, Surabaya 60115, East Java, Indonesia; 3 Department of Veterinary Public Health, Faculty of Veterinary Medicine, Universitas Airlangga, Jl. Dr. Ir. H. Soekarno, Kampus C Mulyorejo, Surabaya 60115, East Java, Indonesia; 4 Study Program of Pharmacy Science, Faculty of Health Science, Universitas Muhammadiyah Surabaya, Jl. Raya Sutorejo No.59, Dukuh Sutorejo, Mulyorejo, Surabaya 60113, East Java, Indonesia; 5 Department of Pathobiology, Faculty of Veterinary and Animal Sciences, Gomal University, RV9W+GVJ, Indus HWY, Dera Ismail Khan 27000, Pakistan

**Keywords:** African swine fever virus, *B646L* gene, genotype II, Indonesia, molecular surveillance, *p72* gene, phylogenetic analysis, swine disease

## Abstract

**Background and Aim:**

African swine fever (ASF) is a highly contagious and often fatal viral disease of domestic pigs caused by African swine fever virus (ASFV), posing a serious threat to global swine production and food security. Since the first confirmed outbreak in Indonesia in 2019, molecular data on circulating strains remain limited, limiting the ability to trace viral transmission and evaluate long-term epidemiological patterns. The *B646L* gene encoding the major capsid protein p72 is widely used for ASFV genotyping and phylogenetic analysis. This study aimed to detect ASFV circulation in Indonesia between 2021 and 2025 and to characterize its phylogenetic relationships using partial sequencing of the *B646L* (p72) gene.

**Materials and Methods:**

A cross-sectional molecular surveillance study was conducted from 2021 to 2025 using samples collected from slaughterhouses and pig-producing areas in several provinces of Indonesia. A total of 272 EDTA blood and serum samples were obtained through routine surveillance and risk-based sampling. DNA was extracted and screened using conventional polymerase chain reaction (PCR) targeting the *B646L* (p72) gene with primer pairs PPA1/PPA2 (257 bp) for detection and P72-U/P72-D (478 bp) for sequencing. Positive amplicons were subjected to bidirectional Sanger sequencing. Sequences were aligned with global reference strains representing ASFV genotypes I–XXIV and analyzed using the Neighbor-Joining method with the Kimura 2-parameter + Gamma model and 1,000 bootstrap replicates.

**Results:**

Seven samples (2.6%) tested positive for ASFV, with detections recorded in North Sumatra, East Nusa Tenggara, Jakarta, and the Medan abattoir. Both blood and serum samples produced clear diagnostic amplicons. All sequence-positive samples clustered within the Eurasian genotype II lineage and showed >99% nucleotide identity with Georgia-2007–derived strains circulating in Asia. Phylogenetic analysis based on partial *B646L* (p72) gene sequences demonstrated strong bootstrap support and revealed no evidence of divergent genotypes, novel variants, or additional lineage introductions, indicating genetic stability of ASFV circulating in Indonesia during the study period.

**Conclusion:**

The findings confirm that ASFV genotype II continues to circulate in Indonesia several years after the initial outbreak and show that the virus has entered an endemic phase marked by low-level yet persistent transmission. Partial sequencing of the *B646L* (p72) gene remains useful for identifying genotypes; however, expanded genomic surveillance is necessary for detailed transmission tracking. Enhancing routine molecular monitoring and integrating phylogenetic analysis into national surveillance programs will be crucial for improving early detection and supporting long-term ASF control strategies.

## INTRODUCTION

African swine fever (ASF) is a highly contagious and often deadly viral disease that affects domestic pigs and wild suids. It is caused by the African swine fever virus (ASFV), a large enveloped double-stranded DNA virus belonging to the family Asfarviridae [1]. ASFV contains more than 150 open reading frames that encode proteins essential for viral replication, evading the host immune system, and adapting to different environmental conditions [2]. The virus is notably stable in the environment, remaining infectious for extended periods in pork products, carcasses, and contaminated fomites, which promotes long-distance and cross-border spread [3]. Although ASF is not zoonotic, its severe clinical signs, including high mortality rates, hemorrhagic lesions, and significant herd losses, cause substantial economic damage to the global swine industry. As a result, the World Organization for Animal Health (WOAH) classifies ASF as a notifiable transboundary animal disease [4].

Since the first confirmed outbreak of African swine fever in North Sumatra in late 2019, ASFV has spread to several provinces in Indonesia, including Deli Serdang, Tana Karo, Bali, East Nusa Tenggara, and parts of Java. The disease has caused widespread pig deaths, disrupted both smallholder and commercial pig farming, and resulted in significant socio-economic losses. Although multiple molecular studies have confirmed the presence of ASFV genotype II in Indonesia, most available data come from initial outbreak investigations or geographically limited studies, leaving important gaps in understanding the long-term circulation and persistence of the virus under endemic conditions.

Globally, ASFV is regarded as one of the most serious viral threats to pig production systems because of its ability to spread quickly through the movement of live animals, contaminated pork products, and human-mediated transport routes [5]. The rapid spread of ASFV genotype II has changed epidemiological patterns in Europe and Asia, mainly due to poor farm-level biosecurity and uncontrolled pig movements [6]. After its introduction into Georgia in 2007, genotype II spread across Eastern Europe, reached China in 2018, and then spread throughout Southeast Asia, including Vietnam, the Philippines, and Indonesia [7]. Once established in pig populations, the virus becomes very difficult to eliminate because there is no effective vaccine and the virus can persist in certain host tissues [8]. These features highlight the urgent need for ongoing molecular surveillance to track viral introduction and evolution [9].

More than a century after ASF was first identified in East Africa, no commercial vaccine or antiviral treatment has been successfully created [10]. Although recovered pigs may develop strong homologous immunity, the complex genome structure of ASFV, its high genetic variability, and its ability to recombine pose major challenges for developing broad-spectrum vaccines and reliable diagnostic tools [11]. Molecular characterization has thus become essential for understanding ASFV epidemiology [12]. The *B646L* gene, which encodes the main capsid protein p72, is the most commonly used genomic marker for ASFV genotyping because of its conserved structure and the established classification system defining 24 genotypes [13]. Worldwide, genotype II is currently the dominant lineage responsible for recent outbreaks across Europe and Asia [14].

Indonesia reported its first confirmed ASF outbreak in North Sumatra in late 2019, with subsequent spread documented in areas such as Deli Serdang, Tana Karo, and East Nusa Tenggara [15]. Molecular investigations conducted by national research institutions have shown that Indonesian ASFV isolates cluster within genotype II and are closely genetically similar to reference strains from Georgia (2007), China (2019), and Vietnam (2019) [16]. However, expanded molecular identification and phylogenetic analysis are still necessary to better understand viral diversity, trace introduction pathways, and support national disease control strategies [17].

Despite the increasing reports of ASF outbreaks in Indonesia since its first detection in 2019, comprehensive molecular surveillance data spanning several years and regions remain limited. Most previous studies were conducted during early outbreak investigations or focused on specific locations, making it hard to determine whether ASFV continues to circulate endemically or if new viral introductions have occurred. Furthermore, available molecular data from Indonesia mainly come from short-term studies and do not adequately reflect the long-term genetic stability of circulating strains. Ongoing monitoring using reliable genomic markers is crucial to understanding the epidemiological dynamics of ASFV, especially in countries where pig production involves extensive animal movement and varied biosecurity practices.

The *B646L* gene, which encodes the major capsid protein p72, is the internationally recognized marker for ASFV genotyping and is commonly used for phylogenetic comparisons of isolates across different regions and time periods. However, limited information is available about the genetic characteristics of ASFV circulating in Indonesia after the initial outbreak, and few studies have assessed virus distribution using samples collected from both slaughterhouses and pig-producing areas under routine surveillance. The lack of long-term, multi-site molecular data hampers the ability to determine whether ASFV genotype II remains the dominant lineage in Indonesia or if genetic variation has developed during ongoing circulation. Consequently, updated molecular identification and phylogenetic analysis are necessary to clarify the current status of ASFV in Indonesia and to aid in the development of evidence-based control strategies.

Therefore, the current study aimed to detect ASFV circulating in Indonesia from 2021 to 2025 and to characterize its phylogenetic relationships using partial sequencing of the *B646L* (p72) gene. This study was planned as a multi-year molecular surveillance project, utilizing samples collected from slaughterhouses and pig-producing regions across several provinces to provide updated information on virus distribution in field conditions. The specific objectives were to identify the presence of ASFV in blood and serum samples through PCR, to obtain nucleotide sequences of the *B646L* gene from positive samples, and to compare these sequences with global reference strains representing ASFV genotypes I–XXIV. Through phylogenetic analysis, this study sought to determine whether the virus circulating in Indonesia remains within the Eurasian genotype II lineage and to evaluate the virus’s genetic stability several years after its introduction. The findings are expected to contribute to enhanced molecular surveillance, support early detection efforts, and provide scientific evidence to strengthen national ASF prevention and control strategies.

## MATERIALS AND METHODS

### Ethical approval

This study was conducted as part of the national African swine fever surveillance program in Indonesia and involved the collection of EDTA blood and serum samples from pigs presented for slaughter and from farms under routine animal health investigation. The sampling activities did not involve experimental infection, treatment trials, or any procedures beyond standard veterinary diagnostic and surveillance practices. Therefore, separate approval from a formal Institutional Animal Care and Use Committee was not required. All field procedures were performed by trained veterinarians and authorized animal health officers in accordance with institutional surveillance protocols, national animal welfare principles, and applicable biosafety regulations for handling suspected transboundary animal disease materials. Sample collection was carried out with minimal distress to animals and only under routine field or slaughterhouse conditions. In addition, all laboratory procedures involving potentially ASFV-positive materials were conducted under biosafety level 2 conditions following national biosafety guidance for veterinary diagnostic laboratories.

### Study period and location

This study was conducted from 2021 to 2025 in several provinces of Indonesia, including North Sumatra, East Nusa Tenggara, and DKI Jakarta. Samples were collected from slaughterhouses and pig-producing areas as part of routine molecular surveillance activities.

### Study design and sampling framework

This study was designed as a cross-sectional molecular surveillance of ASFV conducted from 2021 to 2025. Sampling used a combination of convenience and risk-based methods, targeting both slaughterhouses and pig-producing areas to monitor ASFV circulation along the production and marketing chain. Provinces were chosen based on previous ASF reports, pig population density, and logistical feasibility for routine surveillance activities.

### Sample collection and handling

Whole blood (EDTA) and serum samples were gathered by trained veterinarians and animal health officers in accordance with standard biosafety precautions for ASF-suspected materials. Blood and serum were not always collected from the same animals because samples were obtained opportunistically from slaughterhouses and farms based on field availability. After collection, samples were stored at 4°C in the field and transported to the laboratory within 24–48 h, then kept at −20°C until DNA extraction.

### Sampling

EDTA blood and serum samples were collected using a cross-sectional molecular surveillance design that combined convenience and risk-based sampling from slaughterhouses and pig-producing areas. Samples were obtained from apparently healthy pigs presented for slaughter as well as pigs from farms with recent or suspected ASF history, depending on field access and availability. Metadata recorded for each sample included the sampling site, year of collection, sample code, and specimen type. Sampling procedures followed institutional surveillance guidelines for animal health monitoring, and all activities were performed in accordance with national biosafety and ethical regulations.

### DNA extraction

Total DNA was extracted from 200 µL of EDTA-treated blood or serum using the High Pure PCR Template Preparation Kit (Roche Molecular Biochemicals) following the manufacturer’s instructions. The final elution volume for each sample was 50 µL. Extracted DNA was stored at −20°C until further analysis. A negative extraction control (nuclease-free water) was included in each batch to monitor potential cross-contamination. DNA concentration and purity were not routinely measured because samples were directly subjected to PCR as part of standard ASFV surveillance procedures.

### PCR amplification of the *B646L* (p72) gene

Conventional PCR assays targeting the *B646L* (p72) gene were performed using two primer sets with different analytical purposes. For diagnostic screening, the PPA1/PPA2 primers were used to amplify a 257-bp fragment (PPA1: 5′-AGTTATGGGAAACCCGACCC-3′; PPA2: 5′-CCCTGAATCGGAGCATCCT-3′) [18]. For genotyping and sequencing, the P72-U/P72-D primer pair was used to amplify a 478-bp region (P72-U: 5′-GGCACAAGTTCGGACA TGT-3′; P72-D: 5′’-GTACTGTAACGCAGCACAG-3′) [19].

Each PCR reaction consisted of 1× PCR buffer (50 mM KCl, 10 mM Tris-HCl), 2 mM MgCl_2_, 0.2 mM dNTPs, 0.2 µM of each primer, 0.625 U Taq DNA polymerase, and 2 µL DNA template in a final volume of 25 µL.

Thermal cycling conditions for the PPA1/PPA2 assay were as follows: initial denaturation at 95°C for 10 min, followed by 40 cycles of 95°C for 15 s, 62°C for 30 s, and 72°C for 30 s, with a final extension at 72°C for 7 min. For amplification with the P72-U/P72-D primers, cycling conditions consisted of an initial denaturation at 96°C for 20 s, followed by 35 cycles of 96°C for 12 s, 50°C for 20 s, and 72°C for 25 s, with a final extension at 72°C for 5 min.

A previously confirmed ASFV-positive field sample from the 2019 outbreak in East Nusa Tenggara was used as a positive control in all PCR runs, whereas nuclease-free water served as a no-template negative control. PCR assays were performed in single reactions per sample in accordance with routine surveillance protocols. PCR products were separated on 2% agarose gels stained with ethidium bromide and visualized under ultraviolet illumination to confirm the expected amplicon sizes before sequencing.

### Sequencing and phylogenetic analysis

Amplicons produced using the P72-U/P72-D primers (478 bp) underwent bidirectional Sanger sequencing, which was outsourced to Macrogen (PT. Indolab Utama, Indonesia). Raw chromatogram files were reviewed and trimmed with Chromas software to eliminate low-quality bases, and forward and reverse reads were assembled into consensus sequences. Only sequences with clear and unambiguous peaks throughout most of the target region were kept for further analysis.

Consensus sequences were aligned with a curated global ASFV reference dataset representing genotypes I–XXIV, in which each genotype was represented by one or two well-characterized reference strains. Multiple sequence alignment was performed using the ClustalW algorithm implemented in MEGA X [20]. Phylogenetic trees were constructed using the Neighbor-Joining method with the Kimura 2-parameter model with gamma distribution (K2+G), selected because of its suitability for relatively conserved DNA sequences and its common use in ASFV p72-based genotyping studies. Node reliability was evaluated using 1,000 bootstrap replicates.

The nucleotide sequences generated in this study have been submitted to GenBank and are currently under processing. Accession numbers can be obtained either from GenBank or the corresponding author.

### Data reporting and analysis

PCR and sequencing results were summarized descriptively based on sampling location and specimen type. Positivity rates were calculated by dividing the number of PCR-positive samples by the total samples tested for each site and matrix. All sequence-confirmed ASFV cases were recorded along with sample identifiers, collection sites, specimen types, and related amplicon information. These data were combined with phylogenetic findings to describe the distribution and lineage features of ASFV circulating in Indonesia.

## RESULTS

### PCR detection of ASFV in field samples

A total of 272 clinical specimens, including EDTA blood and serum samples collected from seven provinces between 2021 and 2025, were screened for ASFV using conventional PCR targeting the *B646L* (p72) gene. Overall, seven samples (2.6%) showed positive amplification signals (Table 1). No ASFV DNA was found in samples from Singkawang, West Kalimantan. In contrast, low but consistent positivity was observed in several other regions. One sample from East Nusa Tenggara tested positive (1/18; 5.6%). In North Sumatra, ASFV was detected in both blood and serum samples, with one positive sample each from Deli Serdang blood (1/27; 3.7%), Deli Serdang serum (1/17; 5.9%), and Tana Karo serum (1/25; 4.0%). Additional positive cases were identified at the Medan City abattoir (2/70; 2.9%) and the Kapuk abattoir in Jakarta (1/125; 0.8%). All ASFV-positive samples were identified during the 2023 and 2025 sampling periods, while no positive samples were found in 2021, indicating sustained but low-level circulation of the virus several years after the initial outbreak.

**Table 1:** Detection of ASFV in pig blood (EDTA) and serum samples using PPA1/PPA2 and P72-U/P72-D primer pairs targeting the *B646L* (p72) gene across Indonesian sites, 2021–2025.

Sampling location	Year of collection	Sample code	Sample type	Number of samples	PCR positive (n)	PCR negative (n)	Positivity rate (%)
Singkawang, West Kalimantan	2021	SIND	Blood + EDTA	20	0	20	0.0
East Nusa Tenggara	2023	NTTD	Blood + EDTA	18	1	17	5.6
Deli Serdang, North Sumatra	2023	DSD	Blood + EDTA	27	1	26	3.7
Deli Serdang, North Sumatra	2023	DSD	Serum	17	1	16	5.9
Tana Karo, North Sumatra	2023	TKS	Serum	25	1	24	4.0
Pig slaughterhouse, Medan City	2025	RPMD	Blood + EDTA	70	2	69	2.9
Pig slaughterhouse, Kapuk, DKI Jakarta	2025	RPKD	Blood + EDTA	125	1	124	0.8

ASFV = African swine fever virus, PCR = polymerase chain reaction, EDTA = ethylenediaminetetraacetic acid, bp = base pairs, *B646L* = ASFV major capsid gene encoding p72.

**Figure 1:**
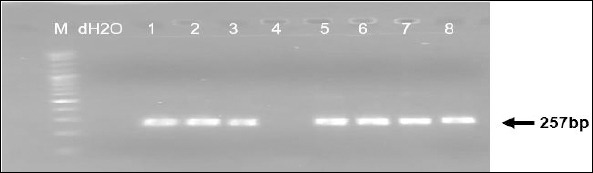
Detection of ASFV using the primer pair PPA1/PPA2 targeting the *B646L* (p72) gene, yielding a 257 bp amplicon. Lane M, 100 bp DNA marker, lane N, negative control (ddH_2_,O), lanes 1–8, field samples from different regions showing the presence of ASFV-specific bands.

**Figure 2:**
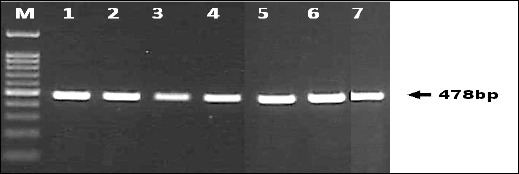
Amplification of a 478 bp fragment of the *B646L* (p72) gene using the primer pair P72-U/P72-D. The amplified product was used for genetic analysis and phylogenetic characterization of ASFV isolates from Indonesia. The 478 bp region represents a conserved but polymorphic segment suitable for genotype differentiation (genotypes I–XXIV).

Both PCR assays produced clear and distinct amplicons of the expected sizes. The diagnostic PPA1/PPA2 primer set consistently generated a 257-bp fragment confirming ASFV detection (Figure 1), while the P72-U/P72-D primers produced the 478-bp fragment used for sequencing and genotyping (Figure 2). Band clarity and uniformity across both EDTA blood and serum samples indicate that these specimen types are suitable for conventional PCR-based ASFV screening. No samples were positive by PPA1/PPA2 but failed amplification with the P72-U/P72-D primers, and no ambiguous or mixed infection bands were observed.

### Sequencing and phylogenetic characterization

All seven PCR-positive samples amplified with the P72-U/P72-D primer pair were successfully sequenced, resulting in high-quality chromatograms suitable for further analysis. Partial *B646L* (p72) gene sequences were aligned and compared with a curated global reference panel that includes ASFV genotypes I–XXIV. Phylogenetic reconstruction using the Neighbor-Joining method with the Kimura 2-parameter + Gamma model and 1,000 bootstrap replicates revealed a consistent and well-supported clustering pattern.

**Figure 3:**
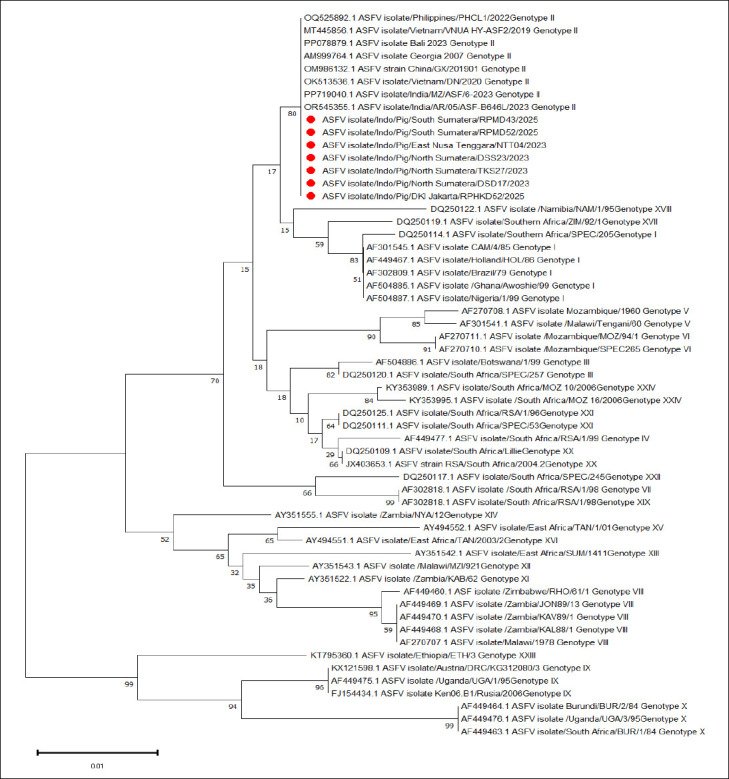
Phylogenetic tree of ASFV based on partial nucleotide sequences of the *B646L* (p72) gene. The tree was constructed using the Neighbor-Joining method with the Kimura 2-parameter + Gamma (K2+G) model implemented in MEGA X®. Topological confidence was evaluated using 1,000 bootstrap replications. Indonesian ASFV isolates (highlighted in red circles) clustered within genotype II and showed close evolutionary relationships with reference isolates from Georgia (2007), Vietnam (2019), China (2019), India (2023), the Philippines (2022), and Bali (2023), indicating circulation of the same evolutionary lineage in Southeast Asia.

Pairwise nucleotide comparisons showed that the Indonesian ASFV sequences shared over 99% identity with Georgia 2007–derived genotype II reference strains from China, Vietnam, the Philippines, and previously reported Indonesian isolates. Only a few synonymous nucleotide substitutions were observed, and no amino acid–changing mutations were found in the analyzed p72 region. All ASFV sequences obtained in this study clustered clearly within the genotype II lineage, with bootstrap values of 80 or higher. These sequences closely grouped with representative isolates of the Georgia 2007–derived Eurasian lineage, including reference strains from Vietnam (2019), China (2019), the Philippines (2022), India (2023), and previously reported Indonesian isolates such as the Bali 2023 strain. No evidence of divergent clustering, unique sub-lineages, or additional genotype introductions was observed (Figure 3). These findings confirm the ongoing circulation of the pandemic Eurasian genotype II strain in the sampled regions of Indonesia.

## DISCUSSION

### Persistence of ASFV circulation under endemic conditions

This study offers one of the first multi-year molecular surveillance datasets of ASFV in Indonesia since its initial introduction in 2019. Unlike early outbreak-focused investigations, the results show low-level yet ongoing circulation of ASFV across geographically distant areas, including production zones and slaughterhouses, suggesting an endemic transmission pattern rather than a single epidemic wave.

Despite the relatively low PCR positivity rate (2.6%) from samples collected between 2021 and 2025, the detection of ASFV DNA across several geographically distant regions, including North Sumatra, East Nusa Tenggara, and Jakarta, indicates ongoing viral circulation within different components of the swine value chain [21]. The detection of ASFV in both EDTA blood and serum further supports the usefulness of these matrices for routine molecular surveillance, consistent with earlier reports showing that ASFV can be reliably detected in various clinical specimens during acute and subacute infections [22].

The relatively low ASFV detection rate (2.6%) aligns with a post-epidemic endemic phase, during which widespread mortality has already occurred, and surviving pig populations, market flows, and improved biosecurity measures lower the likelihood of detecting actively infected animals. Additionally, slaughterhouse-based sampling often includes apparently healthy animals that may carry ASFV at low viral loads or during subclinical infection, further reducing PCR positivity compared to outbreak-driven farm investigations. The detection of ASFV in slaughterhouse samples from Medan and Jakarta emphasizes the epidemiological significance of abattoir-based surveillance as an early warning system. Slaughterhouses process pigs from multiple sources and trading networks, making them key locations for identifying silent ASFV circulation that might not be detected through farm-based surveillance alone.

### Epidemiological relevance and regional transmission pattern

The molecular detection pattern observed in this study aligns with the epidemiological history of ASF outbreaks in Indonesia since its first confirmation in 2019 [23]. The sporadic yet persistent detection in slaughterhouses and pig-producing areas indicates possible underreporting of cases, the presence of subclinical infections, and the movement of infected but clinically normal pigs within local trade systems [24]. Similar patterns have been reported in other Southeast Asian countries, where ASFV continues to circulate endemically several years after its initial introduction due to incomplete depopulation strategies, inadequate biosecurity, and challenges in controlling intra-island and inter-province pig movement [25].

The results of this study align with previous molecular research from Indonesia and nearby Southeast Asian countries, which consistently identified genotype II as the main circulating lineage. However, most earlier studies in Indonesia focused on outbreak investigations or covered limited geographic areas, whereas this study shows ongoing genotype II circulation across multiple provinces over several years under routine surveillance conditions.

### Phylogenetic stability of genotype II based on the *B646L* gene

Phylogenetic analysis of the partial p72 gene revealed that all Indonesian ASFV sequences obtained in this study grouped within genotype II, consistent with previous research conducted by national institutions [26]. Strong bootstrap support and close genetic similarity to reference strains from Georgia (2007), China (2019), Vietnam (2019), and the Philippines (2022) further confirm that the Indonesian outbreaks are part of the broader Eurasian genotype II epidemic that has dominated global ASFV spread over the past decade [3]. No genetic divergence, distinct branching, or emerging sub-lineages were observed among the Indonesian isolates, indicating that genotype II circulating in Indonesia remains genetically conserved [27].

This pattern is typical for ASFV, which generally exhibits low mutation rates in the p72 region, especially over limited geographic and temporal scales [28]. The lack of divergent sub-lineages or unique amino acid substitutions in the p72 region suggests that the ASFV genotype II circulating in Indonesia has remained genetically stable despite extended circulation. This finding contrasts with reports of emerging microvariants in other regions and implies that virus evolution in Indonesia may be limited by minimal selective pressure or the absence of new variant introductions.

### Implications for surveillance and virus introduction pathways

The phylogenetic clustering also supports the hypothesis that ASFV introduction into Indonesia was closely linked to regional dissemination routes from Asia, consistent with patterns reported in neighboring countries [26]. The absence of additional genotypes suggests that Indonesia probably experienced a single primary introduction event followed by local spread rather than multiple independent ones [21]. These findings have significant implications for national surveillance programs. Ongoing genomic monitoring is necessary to identify potential recombination events or new strain introductions, especially given the extensive pig transport networks connecting farms, traders, and slaughterhouses [29].

### Limitations of p72 genotyping and future directions

Although the *B646L* (p72) gene remains the global standard for ASFV genotyping, its limited ability to resolve intra-genotype variation presents an inherent limitation [30]. Future studies that combine p72 sequencing with more variable genomic regions, such as the central variable region (CVR), intergenic regions (IGRs), or whole-genome sequencing, could enable more precise tracing of transmission chains and viral evolution within Indonesia [31]. Expanding sampling coverage to backyard farms, trader networks, and wild boar populations could further enhance understanding of ASFV circulation [32].

A major limitation of this study is the use of partial sequencing of the *B646L* (p72) gene, which provides reliable genotype classification but limited resolution for identifying detailed transmission routes or recent evolutionary changes. Including more variable genomic regions or performing whole-genome sequencing would allow for more accurate reconstruction of transmission pathways and viral adaptation. Nevertheless, p72-based genotyping remains the international standard for ASFV classification and enables direct comparison with historical and global datasets, making it suitable for long-term lineage tracking and surveillance across countries.

### Implications for control strategies

The findings of this study show that Eurasian genotype II ASFV still exists in Indonesia and highlight the need for ongoing molecular surveillance for early detection and outbreak control. The repeated detection of genotype II across different provinces underscores the importance of strengthening biosecurity, improving movement controls, and incorporating genomic data into epidemiological decisions to enable more effective ASF management [33].

## CONCLUSION

This study confirmed the ongoing circulation of ASFV in Indonesia several years after its initial introduction, based on molecular detection and phylogenetic analysis of the *B646L* (p72) gene from samples collected between 2021 and 2025. Although the overall PCR positivity rate was low (2.6%), ASFV DNA was found in multiple geographically distant regions, including North Sumatra, East Nusa Tenggara, Jakarta, and slaughterhouse-derived samples, indicating persistent but low-level viral circulation within different parts of the swine production and marketing chain. Phylogenetic analysis showed that all detected isolates clustered within genotype II and displayed high genetic similarity to Eurasian lineage strains from the Georgia 2007 outbreak, confirming that a single dominant lineage continues to circulate in Indonesia without evidence of new genotype introduction or significant genetic divergence.

From a practical perspective, detecting ASFV in both farm-origin and slaughterhouse samples underscores the importance of combining abattoir-based surveillance with field investigations to enhance early detection of silent virus circulation. The findings also highlight that low detection rates do not necessarily mean the disease is absent but may indicate endemic persistence under conditions of partial immunity, improved biosecurity, and subclinical infection. Therefore, strengthening routine molecular surveillance, especially in high-risk production and trading networks, is crucial for effective ASF monitoring and control.

A major strength of this study is the multi-year, multi-region sampling design, which offers updated information on ASFV circulation under routine surveillance conditions rather than during outbreak-only investigations. However, the study has several limitations. Genotyping relied on partial sequencing of the *B646L* (p72) gene, which is useful for lineage classification but has limited resolution for identifying detailed transmission routes or recent evolutionary changes. Additionally, sampling was opportunistic and dependent on surveillance activities, which may underestimate the true prevalence of infection.

Future research should include larger sample sizes, broader geographic coverage, and additional genomic markers, such as the central variable region, intergenic regions, or whole-genome sequencing, to enable more accurate tracking of virus evolution and transmission pathways. Combining molecular data with epidemiological and movement data will further enhance national ASF surveillance efforts.

In conclusion, the results show that ASFV genotype II remains genetically stable and continues to circulate endemically in Indonesia several years after its introduction. Ongoing molecular monitoring, enhanced biosecurity, and better movement control strategies are vital to support early detection, prevent further spread, and improve long-term ASF control efforts.

## DATA AVAILABILITY

The supplementary data can be made available from the corresponding author upon request.

## AUTHORS’ CONTRIBUTIONS

MS and SS: Conceptualization, supervision, and manuscript drafting. MHE and SR: Data curation and formal analysis. NLPID and ARK: Investigation and visualization. HN and AR: Methodology, data curation, and validation. IS and DAP: Validation, investigation, and data interpretation. BB and WT: Writing – review and editing and data interpretation. All authors have read, reviewed, and approved the final manuscript.
